# Complex relationship between gut microbiota and thyroid dysfunction: a bidirectional two-sample Mendelian randomization study

**DOI:** 10.3389/fendo.2023.1267383

**Published:** 2023-11-10

**Authors:** Xiao Liu, Jingyu Liu, Tongxin Zhang, Qian Wang, Huawei Zhang

**Affiliations:** Department of Ultrasound, Shandong Provincial Hospital Affiliated to Shandong First Medical University, Jinan, Shandong, China

**Keywords:** Mendelian randomization, thyroid dysfunction, hypothyroidism, gut microbiota, thyroid-gut axis

## Abstract

**Background:**

Many studies have reported the link between gut microbiota and thyroid dysfunction. However, the causal effect of gut microbiota on thyroid dysfunction and the changes in gut microbiota after the onset of thyroid dysfunction are not clear.

**Methods:**

A two-sample Mendelian randomization (MR) study was used to explore the complex relationship between gut microbiota and thyroid dysfunction. Data on 211 bacterial taxa were obtained from the MiBioGen consortium, and data on thyroid dysfunction, including hypothyroidism, thyroid-stimulating hormone alteration, thyroxine deficiency, and thyroid peroxidase antibodies positivity, were derived from several databases. Inverse variance weighting (IVW), weighted median, MR-Egger, weighted mode, and simple mode were applied to assess the causal effects of gut microbiota on thyroid dysfunction. Comprehensive sensitivity analyses were followed to validate the robustness of the results. Finally, a reverse MR study was conducted to explore the alteration of gut microbiota after hypothyroidism onset.

**Results:**

Our bidirectional two-sample MR study revealed that the genera *Intestinimonas*, *Eubacterium brachy group*, *Ruminiclostridium5*, and *Ruminococcaceae UCG004* were the risk factors for decreased thyroid function, whereas the genera *Bifidobacterium* and *Lachnospiraceae UCG008* and phyla Actinobacteria and Verrucomicrobia were protective. The abundance of eight bacterial taxa varied after the onset of hypothyroidism. Sensitivity analysis showed that no heterogeneity or pleiotropy existed in the results of this study.

**Conclusion:**

This novel MR study systematically demonstrated the complex relationship between gut microbiota and thyroid dysfunction, which supports the selection of more targeted probiotics to maintain thyroid–gut axis homeostasis and thus to prevent, control, and reverse the development of thyroid dysfunction.

## Introduction

Thyroid dysfunction stands as one of the prevailing endocrine diseases, and hypothyroidism is one of its main types, affecting approximately 5% of the general population (1). Thyroid hormones exert their influence selectively on multiple organs and tissues. Hypothyroidism, a representation of thyroid dysfunction, exhibits strong associations with cardiovascular diseases (2), diabetes (3), and thromboembolism (4), among other conditions. Recent epidemiological studies globally have unveiled a significant correlation between hypothyroidism and elevated mortality rates (5). Hypothyroidism can arise due to factors such as iodine deficiency, medications, radiation therapy, immune system abnormalities, and pregnancy. Once established, reversing hypothyroidism poses significant challenges. Among these triggers, autoimmune hypothyroidism proves especially problematic, given its enduring nature. Hence, exploring autoimmune hypothyroidism demands comprehensive analysis from diverse angles. It necessitates in-depth research to uncover treatments beyond lifelong thyroxine administration. These treatments should not only impede the disease’s progression but also enhance patients’ quality of life significantly.

The human gut microbiota boasts unparalleled diversity and complexity among all human organs, with its composition intricately linked to ethnicity, dietary habits, and geographic location (6). Variations in gut microbiota among healthy individuals from diverse backgrounds pose a challenge in establishing a definitive healthy baseline. This microbiota actively participates in the fundamental physiological functions and diseases within the human body, including nutrient production, metabolic balance, immune response, brain behavior, and inflammatory reactions (7). Moreover, it plays a pivotal role in several endocrine diseases, notably diabetes (8) and polycystic ovary syndrome (9). The evolving recognition of the interconnection between gut microbiota and thyroid function is denoted as the thyroid–gut axis (10, 11). Dysbiosis in the gut microbiota has been evidenced to disrupt the absorption of iodine and to impact the synthesis and release of thyroid hormones (12). Researchers have explored this relationship, achieving compelling results by modulating gut microbiota in mouse models and clinical cases of thyroid dysfunction (13–16). For instance, hyperthyroidism correlates with increased Actinobacteria and decreased Bacteroidetes, whereas specific strains of *Bifidobacterium* and *Lactobacillus* were found to interact with human autoantibodies, disrupting thyroid function (14, 17). Clinical study has also linked severe hypothyroidism to higher instances of small intestinal bacterial overgrowth, contributing to gastrointestinal symptoms (18).

Although these studies have indicated associations between gut microbiota and thyroid dysfunction, causality remains elusive in traditional observational studies due to confounding and reverse causation, which can lead to biased conclusions. A bidirectional two-sample Mendelian randomization (MR) study presents an innovative method for studying the causal effects between environmental exposures and diseases, or between two diseases, akin to a randomized controlled trial (RCT) (19). MR employs single-nucleotide polymorphisms (SNPs) in genes as instrumental variables (IVs), effectively circumventing confounding factors, and provides statistically significant causal effects by combining SNPs from genome-wide association studies (GWASs) of exposure and outcome (20, 21). It allows for better modeling of random assignment, thereby reducing bias in observational studies and better inferring causality. Although MR has been applied broadly to investigate the gut microbiota’s role in diseases like cancers and autoimmune disorders, its application to thyroid dysfunction remains unexplored (22–24). Therefore, we conducted this bidirectional two-sample MR study utilizing thyroid function–related GWAS data from the international consortia, including the MiBioGen consortium, FinnGen consortium, MRC IEU OpenGWAS project, and ThyroidOmics. In this study, the application of two-sample MR involved the utilization of two distinct, independent European samples for analysis. This approach was employed to mitigate the potential biases associated with a single sample, thereby enhancing the reliability and generalizability of our findings. Furthermore, we used the bidirectional MR that simultaneously considered two different causal directions between the gut microbiota and autoimmune hypothyroidism, thus providing for a more nuanced understanding of the bidirectional causal relationship between them. This comprehensive MR analysis, to our knowledge, marks the first exploration of the association between gut microbiota and thyroid function. These results can determine the bidirectional causal effect between the gut microbiota and thyroid dysfunction and thus guide the regulation of the thyroid–gut axis.

## Methods

### Study design

**Figure 1:**
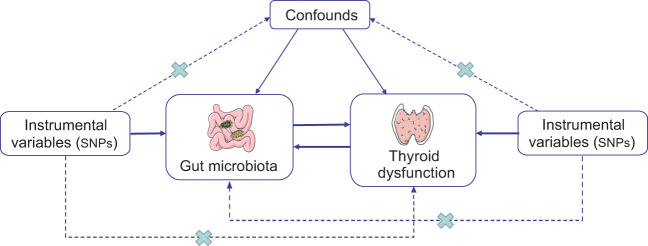
Schematic representation of the bidirectional two-sample MR study. The arrows show three key assumptions for instrumental variables commonly required in MR studies, and the crosses represent possible ways that the assumptions could be violated. SNP, single-nucleotide polymorphism.

The study’s flowchart is depicted in **Figure 1**. To investigate the interplay between gut microbiota and thyroid dysfunction, we selected gut microbiota as the exposure and thyroid function–related factors as outcomes. MR study adhered to three key assumptions: (1) IVs selected from datasets were linked to exposure; (2) they were unrelated to unidentified exposure confounders; and (3) they influenced outcomes exclusively through exposure pathways (25). SNPs served as valid IVs in the MR study to evaluate the bidirectional causal relationship between exposure and outcome.

### Data sources

All data utilized in this study were sourced from diverse GWASs. Regarding thyroid function, the GWASs focused on hypothyroidism, thyroid-stimulating hormone (TSH), thyroxine deficiency, and thyroid peroxidase antibodies (TPOAb) positivity except for gut microbiota data. For the gut microbiota analysis, we extracted the relevant genetic IVs from a comprehensive GWAS dataset provided by the MiBioGen consortium. This dataset was composed of 18,340 participants, primarily of European descent (n = 13,266), and comprised a total of 5,717,754 SNPs after imputation (21). The MiBioGen GWAS amalgamated outcomes from 16S ribosomal RNA gene sequencing effectively eliminated potential batch effects. In total, the dataset contained information on 211 taxa, including 131 genera, 35 families, 20 orders, 16 classes, and nine phyla. As for the thyroid function investigation, the genetic IVs were acquired from the FinnGen consortium (26), MRC IEU OpenGWAS project (27), and ThyroidOmics consortium (28), respectively. The details of each database are summarized in **Table 1**.

**Table 1:** Data sources used in the bidirectional two-sample MR study.

Data	Trait	Year	Consortium	Ancestry	Sample size	Number of SNPs
Gut microbiomes	Gut microbiota abundance	2021	MiBioGen consortium	European	13,266	5,717,754
Hypothyroidism	Hypothyroidism, strict autoimmune	2021	FinnGen consortium	European	198,472	16,380,353
TSH alteration	Thyroid-stimulating hormone	2018	MRC IEU OpenGWAS project	European	3,301	10,534,735
Thyroxine deficiency	Medication code: levothyroxine sodium	2018	MRC IEU OpenGWAS project	European	462,933	9,851,867
TPOAb positivity	Thyroid peroxidase antibodies’ positivity	2014	ThyroidOmics consortium	European	18,297	10,485,757

### Instrumental variables selection

To infer an accurate and realistic bidirectional causal effect between gut microbiota and hypothyroidism risk, we performed a rigorous quality control to select the best IVs. First, we screened SNPs associated with each bacterial taxon using a mild p-value of 1 × 10^−5^. Then, linkage disequilibrium analysis was used to select independent IVs for each bacterial taxon to prevent biased causal estimates. We selected the most suitable parameters for clustering (R^2^ < 0.01 and clustering distance = 500 kb) to evaluate the linkage disequilibrium among the included SNPs. The population was framed as European. Missing SNPs from bacterial taxa in thyroid dysfunction–related datasets were replaced with proxy SNPs (R^2^ > 0.8). Palindromic SNPs were excluded to prevent coding distortions. The F-statistic was calculated to detect the presence of weak IV instrument bias. When the F-statistic is greater than 10, it indicates that there is no weak instrument bias.

### MR analysis

Five widely used MR methods were employed to detect the bidirectional causal relationships between exposure and outcome, encompassing inverse variance weighting (IVW), weighted median, MR-Egger, weighted mode, and simple mode (29–32). IVW method estimates the causal effect of exposure on the outcome by integrating ratio estimates for each SNP, and it was chosen as the primary method because it can provide a robust and unbiased causal effect when no polymorphism or heterogeneity is found (33). The weighted median method correctly estimates causality when up to 50% of the IVs are invalid (30). MR-Egger method is based on the assumption that instrument strengths are independent of direct effects and thereby allows calibration of pleiotropy and calculation of causal inferences, even if all genetic variants are polymorphic (34). If this assumption is violated, then the weighted model method would have greater power to detect causal effects and produce less bias than the MR-Egger method (34). Finally, the simple model method is an unweighted model of the experienced density function for causal estimation (35). A positive causal effect was affirmed if the IVW results were significant (p < 0.05) and beta values from other methods concurred in direction. Wald radio method was used to estimate the causal effect of exposure on the outcome when there was only one SNP, which was the simplest calculation method (36). Then, we further visualized the results of the five MR methods. The bidirectional causal effect was expressed as an odds ratio (OR) calculated from MR analysis. Notably, to obtain more IVs, we did not reach the traditionally strict significance threshold (p < 5 × 10^−8^) for exposure to gut microbiota. Thus, a false discovery rate (FDR) was corrected for multiple comparisons by the Benjamin–Hochberg method to limit the possibility of false positives, and the threshold was set at 0.05. The bidirectional causal effect was considered significant when p < 0.05 and FDR < 0.05 (37).

### Sensitivity analyses

Several basic sensitivity analyses were used to validate the results. Cochran’s Q statistic was employed to assess heterogeneity among IVs. The heterogeneity should be noted if it exists between different IVs (p < 0.05) (38). Horizontal pleiotropy indicates that IVs are associated with the outcome through pathways other than causal effects, potentially leading to false positive results (p < 0.05). MR pleiotropy residual sum and outlier (MR-PRESSO) analysis was also used to validate the potential pleiotropy of the direct effect between the selected IVs and the outcome. Subsequently, we applied the leave-one-out method to exclude each SNP from the IVs, evaluatingwhether individual SNPs significantly influenced causal effects using the IVW method. All the above analyses were done using two-sample MR and MR-PRESSOR R packages (35, 39).

### Reverse MR analysis

To investigate alterations of gut microbiota following the onset of hypothyroidism, we conducted a reverse MR analysis, treating hypothyroidism as the exposure and gut microbiota as the outcome. The threshold of significance level for IVs was adjusted to be more accurate (p < 5 × 10^−8^). The procedure for the reverse MR analysis was the same as the MR analysis described above.

## Results

### Causal effects of gut microbiota on thyroid dysfunction

A total of 14,405 SNPs associated with the gut microbiota were meticulously curated following a rigorous screening process. Through robust linkage disequilibrium clustering and coordination, each bacterial taxon was linked to a varied range of IVs, spanning from 3 to 22. Notably, the F-statistic for all SNPs surpassed 10, indicating the absence of weak instrumental bias in the dataset.

**Figure 2:**
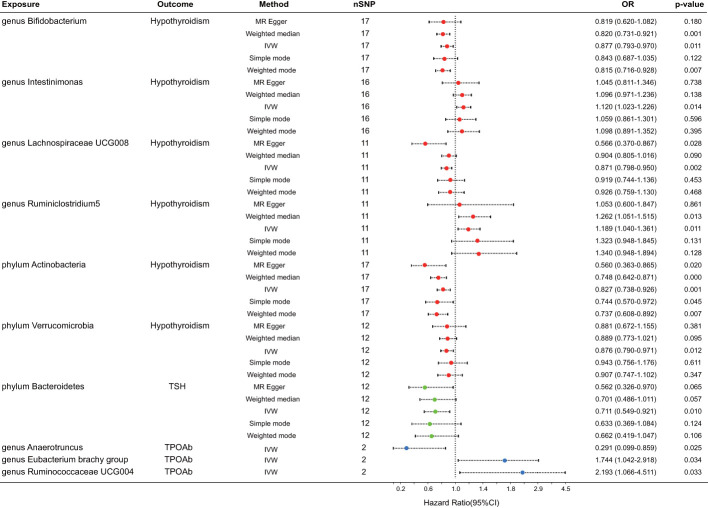
Forest plot for the causal effect of gut microbiota on thyroid dysfunction by the five MR methods. The error bar represents the 95% confidence interval of hazard ratio. The red dot represents an outcome of hypothyroidism, the green represents an outcome of TSH alteration, and the blue represents an outcome of TPOAb positivity. nSNP, number of SNPs; OR, odds ratio; MR, Mendelian randomization; IVW, inverse variance weighted.

**Figure 3:**
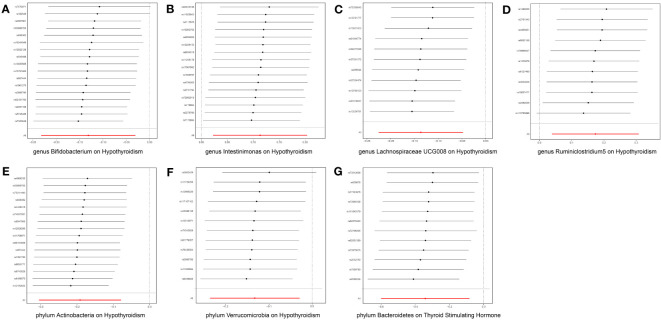
Leave-one-out plots for the causal effect of gut microbiota on thyroid dysfunction. The red line is the random effect of the IVW method, and the error bar represents the 95% confidence interval with the IVW method. **(A)** Genus *Bifidobacterium*, **(B)** genus *Intestinimonas*, **(C)** genus *Lachnospiraceae UCG008*, **(D)** genus *Ruminiclostridium5*, **(E)** phylum Actinobacteria, **(F)** phylum Verrucomicrobia, and **(G)** phylum Bacteroidetes.

After FDR correction, a meticulous analysis targeted three phyla and seven genera of bacteria taxa, revealing their causal effects on thyroid dysfunction via MR analysis. Six were linked to hypothyroidism, one to alterations in TSH levels, and three to TPOAb positivity (**Figure 2**). The IVW estimate pointed toward the genera *Intestinimonas* (OR = 1.120, p = 0.014) and *Ruminiclostridium5* (OR = 1.189, p = 0.011) as the risk factors for hypothyroidism. In contrast, genera *Bifidobacterium* (OR = 0.877, p = 0.011) and *Lachnospiraceae UCG008* (OR = 0.871, p = 0.002) and phyla Actinobacteria (OR = 0.827, p = 0.001) and Verrucomicrobia (OR = 0.876, p = 0.012) emerged as the protective factors against hypothyroidism. Moreover, phylum Bacteroidetes displayed an association with reduced TSH levels (OR = 0.711, p = 0.010). Three bacterial genera exhibited potential causal effects on TPOAb positivity: genus *Anaerotruncus* (OR = 0.291, p = 0.025) reduced TPOAb positivity, whereas genera *Eubacterium brachy group* (OR = 1.744, p = 0.034) and *Ruminococcaceae UCG004* (OR = 2.193, p = 0.033) increased TPOAb positivity, as indicated by the IVW method. Intriguingly, no bacteria demonstrated a significant causal relationship with thyroxine deficiency. Rigorous sensitivity analyses revealed the absence of heterogeneity and pleiotropy in the identified causal effects (**Supplementary Table 1**). Furthermore, the leave-one-out method demonstrated the absence of outlier SNPs in all selected bacteria taxa. Regrettably, this method could not be performed due to the limited number of SNPs associated with TPOAb as the outcome (**Figure 3**).

### Causal effects of thyroid dysfunction on the gut microbiota

**Figure 4:**
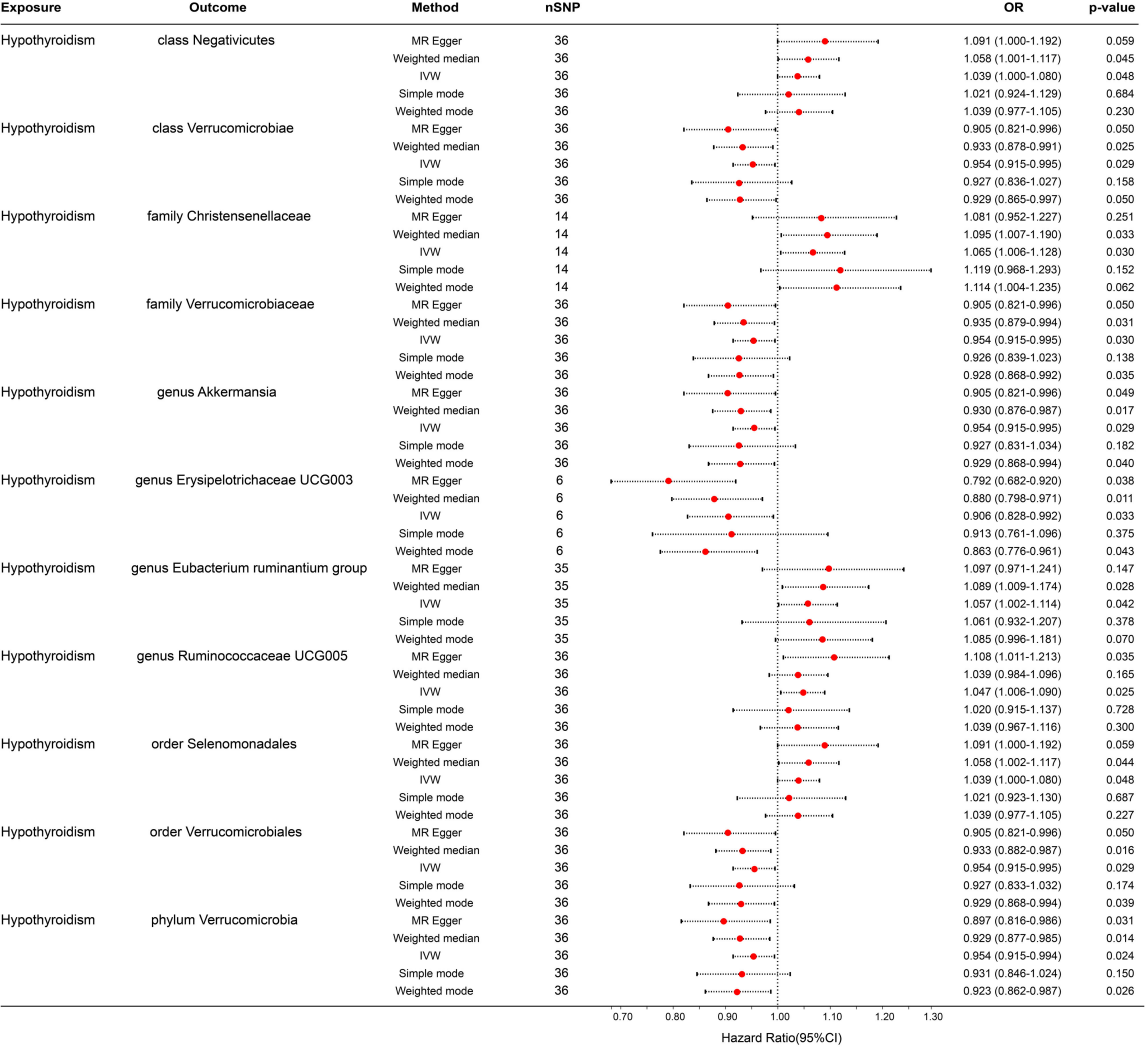
Forest plot for the causal effect of hypothyroidism on gut microbiota by the five MR methods. The error bar represents the 95% confidence interval of the hazard ratio. nSNP, number of SNPs; OR, odds ratio; MR, Mendelian randomization; IVW, inverse variance weighted.

**Figure 5:**
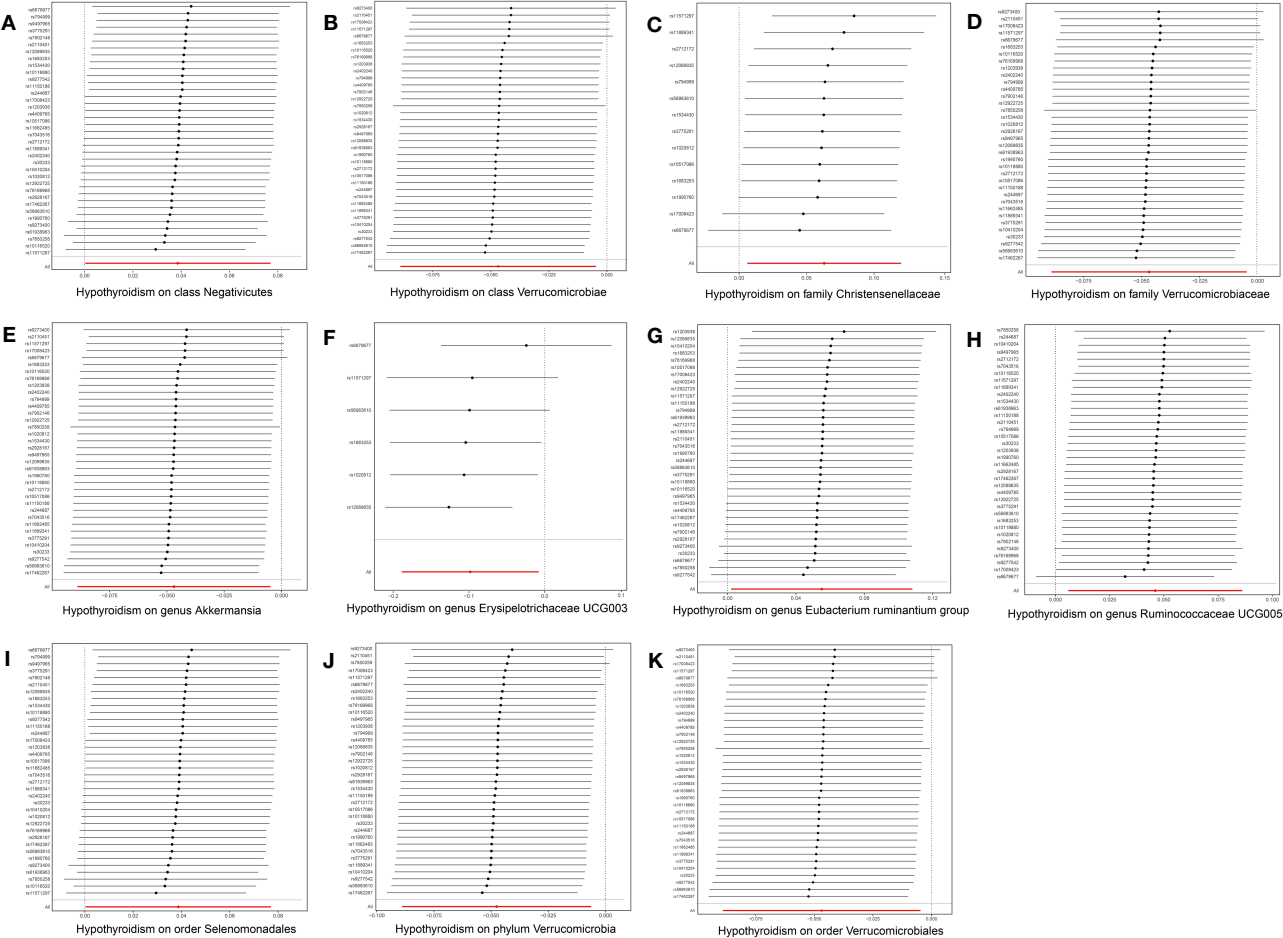
Leave-one-out plots for the causal effect of hypothyroidism on gut microbiota. The red line is the random effect of the IVW method and the error bar represents the 95% confidence interval with the IVW method. **(A)** Class Negativicutes, **(B)** class Verrucomicrobiae, **(C)** family Christensenellaceae, **(D)** family Verrucomicrobiaceae, **(E)** genus *Akkermansia*, **(F)** genus *Erysipelotrichaceae UCG003*, **(G)** genus *Eubacterium ruminantium group*, **(H)** genus *Ruminococcaceae UCG005*, **(I)** order Selenomonadales, **(J)** order Verrucomicrobiales, and **(K)** phylum Verrucomicrobia.

In the reverse MR analysis, 56 SNPs linked with hypothyroidism were identified. Each bacterial taxon was linked to a minimum of 3 and a maximum of 36 IVs, all exhibiting robustness (F-statistic > 10). Applying the IVW method in the MR analysis, it was observed that class Negativicutes (OR = 1.039, p = 0.048), family Christensenellaceae (OR = 1.065, p = 0.030), genera *Eubacterium ruminantium group* (OR = 1.057, p = 0.042) and *Ruminococcaceae UCG005* (OR = 1.047, p = 0.025), and order Selenomonadales (OR = 1.039, p = 0.048) exhibited upregulation following the onset of hypothyroidism. Conversely, class Verrucomicrobiae (OR = 0.954, p = 0.029), family Verrucomicrobiaceae (OR = 0.954, p = 0.024), order Verrucomicrobiales (OR = 0.954, p = 0.029), phylum Verrucomicrobia (OR = 0.954, p = 0.024), and genera *Akkermansia* (OR = 0.954, p = 0.029) and *Erysipelotrichaceae UCG003* (OR = 0.906, p = 0.033) were downregulated subsequent to the onset of hypothyroidism (**Figure 4**). Rigorous sensitivity analyses confirmed the absence of heterogeneity or horizontal pleiotropy in the aforementioned causal effects (**Supplementary Table 2**). In addition, leave-one-out analysis did not reveal any SNPs driving the causal effect of hypothyroidism on gut microbiota (**Figure 5**).

## Discussion

Thyroid dysfunction has emerged as a global public health concern. Advancements in scientific inquiry have delved deeper into gut microbiota research, particularly in its connection to thyroid function via the thyroid–gut axis, a burgeoning area of interest (1, 40). Hypothyroidism, a prevalent condition stemming from thyroid dysfunction, exhibits multifaceted and poorly understood causes. Beyond external causes such as iodine deficiency, medications, and surgery, autoimmune origins constitute the bulk of primary hypothyroidism, posing significant challenges for explanation and management (41). Gut microbiota serves as the linchpin for stable intestinal lymphoid tissue function, acting as a vital shield in immune homeostasis, enhancing tolerance to autoantigens and non-pathogenic non-autoantigens (11). The interplay between gut microbiota and the host’s innate and adaptive immunity potentially influences the susceptibility to autoimmune thyroid disease (42, 43). Moreover, reduced intestinal motility in patients with hypothyroidism might disrupt intestinal substrate utilization and physicochemical conditions, culminating in gut microbiota dysbiosis exacerbating the condition or giving rise to complications (44). To address these intricacies, we conducted a comprehensive bidirectional two-sample MR study utilizing multiple GWAS datasets. This investigation aimed to elucidate the bidirectional causal effects links between gut microbiota and thyroid dysfunction, shedding light on the pathogenesis of autoimmune hypothyroidism and informing strategies for prevention, delay, and reversal of thyroid dysfunction–associated health conditions.

Numerous clinical trials have identified disparities in gut microbiota composition between thyroid dysfunction patients and healthy populations. However, establishing the bidirectional causal relationship between gut microbiota alterations and thyroid dysfunction remained elusive. For instance, a cross-sectional study encompassing 97 cases revealed significantly lower levels of *Alistipes*, *Lachnospiraceae*, *Intestinimonas*, *Ruminococcus*, and *Subdoligranulum* in hypothyroid patients, whereas *Phascolarctobacterium* and Bacteroidetes were more abundant (45). Another study comparing 29 hypothyroid patients with 11 healthy individuals highlighted a higher prevalence of Actinobacteria and *Enterobacteriaceae* and significantly diminished counts of *Bifidobacteria* and *Ruminococcaceae* in the hypothyroid group (46). Our MR analysis not only validated these findings but also established the bidirectional causal relationship between altered gut microbiota and diminished thyroid function. Notably, the genera *Intestinimonas* and *Ruminiclostridium5* were linked to reduced thyroid function, whereas the genera *Bifidobacterium* and *Lachnospiraceae UCG008* and phylum Actinobacteria mitigated this decline. Furthermore, only after the onset of hypothyroidism did the genera *Eubacterium ruminantium group*, *Ruminococcaceae UCG005*, and *Erysipelotrichaceae UCG003* exhibit changes (47, 48). Previous controlled studies also revealed a significant decrease in Bacteroidetes in hyperthyroid patients with elevated TSH levels, with MR analysis corroborating Bacteroidetes’ role in decreasing TSH (49). In an RCT, decreased *Eubacterium* post-probiotic intervention ameliorated complications in patients not treated with radioiodine after thyroidectomy, aligning with our study’s identification of Eubacterium as a risk factor for TPOAb positivity (50).

Several mechanisms underpinning gut microbiota’s impact on thyroid function via the thyroid–gut axis have been elucidated. The equilibrium between pathogenic and probiotic bacteria is pivotal for maintaining gut barrier function. *Bifidobacterium*, a widely used probiotic, confers several physiological benefits to humans, rendering it a protective genus against hypothyroidism in our study (51). In addition, phylum Actinobacteria and genus *Lachnospiraceae UCG008*, both protective in this study, are implicated in human sugar and protein metabolism. Phylum Actinobacteria participates in the biosynthesis of phenylalanine, tyrosine, and tryptophan, whereas *Lachnospiraceae UCG008* is associated with alanine, aspartate, and glutamate metabolism (52–54). Our results also identified *Ruminiclostridium5* as a risk for promoting hypothyroidism. Although limited information is available on *Ruminiclostridium5*, its increased abundance is linked to systemic inflammation and a negative correlation with secondary and conjugated bile acids (55, 56). Consistent with prior studies, phylum Verrucomicrobia emerged as a protective factor against hypothyroidism in our result. This phylum comprises diverse beneficial bacteria for the gut, whose outer membrane proteins effectively safeguard interactions with other cells (57). Genus *Akkermansia*, a member of the phylum Verrucomicrobia, plays a significant role in enhancing host metabolic function and immune responses. Intriguingly, its abundance significantly decreases after hypothyroidism onset (58). Despite these insights, in-depth *in vivo* and *in vitro* experiments are imperative to explore the effects and mechanisms of gut microbiota as delineated in our findings.

The bidirectional two-sample MR study offers significant advantages in investigating the bidirectional causal relationship between gut microbiota and hypothyroidism. First, the sample size of clinical trials often lacks the representativeness necessary for generalizability. In contrast, our study utilized gut microbiome data from 13,266 samples. Thyroid function–related data were sourced from four datasets in three databases, encompassing hundreds of thousands of samples, each mutually exclusive. This vast dataset enhances the present results’ representativeness and credibility. Moreover, IV analysis grounded in effectively mitigates confounding factors and eliminates the outcome’s interference with the exposure’s reverse effect. Utilizing the MR-PRESSO approach and a comprehensive set of sensitivity analyses eliminates study pleiotropy and heterogeneity. The non-duplication of GWAS datasets for both exposure and outcome samples significantly reduces and avoids bias. However, it is crucial to acknowledge the study’s limitations. Gut microbiota data were derived from the GWAS meta-analysis rather than raw data, precluding subgroup analyses. In addition, the study’s inclusion was limited to individuals of European ancestry, restricting the applicability of our findings to broader populations. Furthermore, the lowest taxonomic level for gut bacteria is the genus, precluding an in-depth exploration of gut microbiota’s causal effects on hypothyroidism at the species level. Notably, some results, with a limited number of SNPs, necessitate cautious interpretation. It is pivotal to underscore that RCTs remain the gold standard for treatment development and establishing causal relationships in biological contexts. They offer unparalleled control and evidence. Mendelian randomization analysis serves as a potent supplementary tool, especially in scenarios where conducting RCTs proves challenging.

## Conclusions

In summary, this study firstly and comprehensively provides evidence to support the effects of various gut microbiota on thyroid dysfunction, including hypothyroidism, TSH, thyroxine, and TPOAb, and further reveals alterations in gut microbiota following hypothyroidism. Genera *Intestinimonas*, *Eubacterium brachy group*, *Ruminiclostridium5*, and *Ruminococcaceae UCG004* are found to be risk factors for decreased thyroid function, whereas genera *Bifidobacterium* and *Lachnospiraceae UCG008* and phyla Actinobacteria and Verrucomicrobia were protective. Eight types of gut microbiota are thought to show altered abundance after the onset of hypothyroidism. This bidirectional two-sample MR study provides fairly strong evidence for the thyroid–gut axis theory to select more targeted probiotics to reverse the disturbed immune system and control the progression of thyroid dysfunction.

## Data availability statement

The datasets presented in this study can be found in online repositories. The names of the repository/repositories can be found in the article.

## Author contributions

XL: Data curation, Methodology, Software, Writing – original draft, Investigation. JL: Methodology, Writing – original draft, Formal Analysis. TZ: Software, Writing – original draft, Data curation. QW: Conceptualization, Validation, Writing – review & editing, Funding acquisition, Methodology, Supervision, Visualization. HZ: Supervision, Writing – review & editing, Project administration.
