# Supplementary material for: Complex relationship between gut microbiota and thyroid dysfunction: a bidirectional two-sample Mendelian randomization study
Source: Front Endocrinol (Lausanne). 2023 Nov 10;14:1267383. doi: 10.3389/fendo.2023.1267383 (PMC10667917; doi:10.3389/fendo.2023.1267383)
Supplement: Supplementary file 1 [file Table_1.docx]

Supplementary Material

**Supplementary Table 1.** The causal effect of gut microbiota on thyroid dysfunction according to the IVW method.

| Exposure | Outcome | SNP | β | SE | OR | P-value | FDR | P _Heterogeneity_ | P _Pleiotropy_ |
| --- | --- | --- | --- | --- | --- | --- | --- | --- | --- |
| class Actinobacteria | Hypothyroidism | 18 | -0.105 | 0.050 | 0.900 | 0.036 | 0.060 | 0.197 | 0.860 |
| family Alcaligenaceae | Hypothyroidism | 14 | -0.138 | 0.061 | 0.871 | 0.025 | 0.123 | 0.970 | 0.841 |
| family Enterobacteriaceae | Hypothyroidism | 7 | -0.211 | 0.080 | 0.810 | 0.008 | 0.081 | 0.829 | 0.801 |
| genus Bifidobacterium | Hypothyroidism | 17 | -0.131 | 0.051 | 0.877 | 0.011 | 0.049 | 0.076 | 0.610 |
| genus Intestinimonas | Hypothyroidism | 16 | 0.113 | 0.046 | 1.120 | 0.014 | 0.049 | 0.799 | 0.576 |
| genus Lachnospiraceae UCG008 | Hypothyroidism | 11 | -0.139 | 0.045 | 0.871 | 0.002 | 0.024 | 0.335 | 0.075 |
| genus Ruminiclostridium5 | Hypothyroidism | 11 | 0.174 | 0.069 | 1.189 | 0.011 | 0.049 | 0.691 | 0.672 |
| genus Ruminococcaceae UCG011 | Hypothyroidism | 8 | -0.075 | 0.036 | 0.928 | 0.036 | 0.099 | 0.884 | 0.729 |
| order Enterobacteriales | Hypothyroidism | 7 | -0.211 | 0.080 | 0.810 | 0.008 | 0.081 | 0.829 | 0.801 |
| order Mollicutes RF9 | Hypothyroidism | 14 | 0.119 | 0.050 | 1.127 | 0.016 | 0.081 | 0.275 | 0.954 |
| phylum Actinobacteria | Hypothyroidism | 17 | -0.190 | 0.058 | 0.827 | 0.001 | 0.005 | 0.247 | 0.091 |
| phylum Verrucomicrobia | Hypothyroidism | 12 | -0.133 | 0.053 | 0.876 | 0.012 | 0.029 | 0.676 | 0.961 |
| class Bacteroidia | TSH | 15 | -0.244 | 0.122 | 0.783 | 0.045 | 0.113 | 0.406 | 0.758 |
| genus Actinomyces | TSH | 7 | 0.255 | 0.115 | 1.290 | 0.027 | 0.166 | 0.905 | 0.706 |
| genus Eubacterium coprostanoligenes group | TSH | 13 | 0.279 | 0.136 | 1.322 | 0.041 | 0.166 | 0.809 | 0.571 |
| genus Ruminococcus gnavus group | TSH | 12 | 0.196 | 0.097 | 1.216 | 0.043 | 0.166 | 0.231 | 0.648 |
| order Bacteroidales | TSH | 15 | -0.244 | 0.122 | 0.783 | 0.045 | 0.113 | 0.406 | 0.758 |
| phylum Bacteroidetes | TSH | 12 | -0.341 | 0.132 | 0.711 | 0.010 | 0.048 | 0.576 | 0.362 |
| genus Oxalobacter | Thyroxine deficiency | 11 | 0.003 | 0.001 | 1.003 | 0.018 | 0.137 | 0.641 | 0.256 |
| genus Phascolarctobacterium | Thyroxine deficiency | 10 | 0.004 | 0.002 | 1.004 | 0.036 | 0.153 | 0.840 | 0.638 |
| genus Ruminococcaceae NK4A214 group | Thyroxine deficiency | 14 | 0.005 | 0.002 | 1.005 | 0.018 | 0.137 | 0.749 | 0.301 |
| family Lachnospiraceae | TPOab | 4 | 0.661 | 0.324 | 1.937 | 0.041 | 0.103 | 0.610 |  |
| genus Anaerotruncus | TPOab | 2 | -1.234 | 0.552 | 0.291 | 0.025 | 0.034 | 0.963 |  |
| genus Eubacterium brachy group | TPOab | 2 | 0.556 | 0.263 | 1.744 | 0.034 | 0.034 | 0.438 |  |
| genus Ruminococcaceae UCG004 | TPOab | 2 | 0.785 | 0.368 | 2.193 | 0.033 | 0.034 | 0.445 |  |

**Supplementary Table 2.** The causal effect of hypothyroidism on gut microbiota according to the IVW method.

| exposure | outcome | SNP | β | SE | OR | P-value | P _Heterogeneity_ | P _Pleiotropy_ |
| --- | --- | --- | --- | --- | --- | --- | --- | --- |
| Hypothyroidism | class Negativicutes | 36 | 0.039 | 0.020 | 1.039 | 0.048 | 0.236 | 0.144 |
| Hypothyroidism | class Verrucomicrobiae | 36 | -0.047 | 0.021 | 0.954 | 0.029 | 0.237 | 0.919 |
| Hypothyroidism | family Christensenellaceae | 14 | 0.063 | 0.029 | 1.065 | 0.030 | 0.800 | 0.526 |
| Hypothyroidism | family Verrucomicrobiaceae | 36 | -0.047 | 0.021 | 0.954 | 0.030 | 0.235 | 0.919 |
| Hypothyroidism | genus Akkermansia | 36 | -0.047 | 0.021 | 0.954 | 0.029 | 0.236 | 0.921 |
| Hypothyroidism | genus Erysipelotrichaceae UCG003 | 6 | -0.098 | 0.046 | 0.906 | 0.033 | 0.105 | 0.263 |
| Hypothyroidism | genus Eubacterium ruminantium group | 35 | 0.055 | 0.027 | 1.057 | 0.042 | 0.507 | 0.570 |
| Hypothyroidism | genus Ruminococcaceae UCG005 | 36 | 0.046 | 0.020 | 1.047 | 0.025 | 0.188 | 0.172 |
| Hypothyroidism | order Selenomonadales | 36 | 0.039 | 0.020 | 1.039 | 0.048 | 0.236 | 0.144 |
| Hypothyroidism | order Verrucomicrobiales | 36 | -0.047 | 0.021 | 0.954 | 0.029 | 0.237 | 0.919 |
| Hypothyroidism | phylum Verrucomicrobia | 36 | -0.048 | 0.021 | 0.954 | 0.024 | 0.169 | 0.922 |
